# Maintaining Access to Orthopaedic Surgery During Periods of Operating Room Resource Constraint: Expanded Use of Wide-Awake Surgery During the COVID-19 Pandemic

**DOI:** 10.5435/JAAOSGlobal-D-20-00100

**Published:** 2020-12-15

**Authors:** Justin J. Turcotte, Benjamin M. Petre, Christopher M. Jones, Jeffrey M. Gelfand

**Affiliations:** From the Department of Orthopedics, Anne Arundel Medical Center, Annapolis, MD.

## Abstract

Wide-awake local anesthesia no tourniquet (WALANT) presents a nonstandard anesthetic approach initially described for use in hand surgery that has gained interest and utilization across a variety of orthopaedic procedures. In response to operating room resource constraints imposed by the COVID-19 pandemic, our orthopaedic service rapidly adopted and expanded its use of WALANT.

**Methods:**

A retrospective review of 16 consecutive cases performed by 7 surgeons was conducted. Patient demographics, surgical details, and perioperative outcomes were assessed. The primary end point was WALANT failure, defined as intraoperative conversion to general anesthesia.

**Results:**

No instances of WALANT failure requiring conversion to general anesthesia occurred. In recovery, one patient (6%) required narcotics for pain control, and the average postoperative pain numeric rating scale was 0.6. The maximum pain score experienced was 4 in the patient requiring postoperative narcotics. The average time in recovery was 42 minutes and ranged from 8 to 118 minutes.

**Conclusion:**

The WALANT technique was safely and effectively used in 16 cases across multiple orthopaedic subspecialties, including three procedures not previously described in the literature. WALANT techniques hold promise for use in future disaster scenarios and should be evaluated for potential incorporation into routine orthopaedic surgical care.

Standard methods of general and regional anesthesia, which remain the mainstay of treatment in orthopaedic surgery, are not without inherent risks and costs.^1^ Wide-awake local anesthesia no tourniquet (WALANT) presents an alternative anesthetic approach initially described for use in hand surgery that has gained interest and utilization across a variety of orthopaedic procedures.^1,2,3,4,5^ The technique, which does not require the presence of an anesthesiologist, relies on preincision injection of local anesthetic into the surgical site for pain control and the vasoconstrictive effect of epinephrine to mitigate operative field bleeding in lieu of a tourniquet.^2^ This use of epinephrine to achieve intraoperative hemostasis is the main differentiator between WALANT and the use of other peripheral nerve blocks (PNBs) performed without sedation. Wide-awake PNB without sedation is not routinely performed for orthopaedic extremity surgery because of the discomfort associated with tourniquet use, which is still required in the absence of a pharmacologic means of achieving hemostasis. An additional benefit of WALANT over standard PNB is that it does not require ultrasonography guidance for the injection of the local anesthetic, therefore reducing the learning curve required to use the technique. Proponents of WALANT describe a variety of advantages over standard anesthesia including increased safety from elimination of sedation and its associated adverse effects, decreased need for preoperative testing and medical clearance, elimination of tourniquet pain, elimination of the need for fasting or a driver for patients, and decreased surgical time for anesthesia induction.^2,6,7^ Beyond these clinical benefits, WALANT has been demonstrated to enhance the cost effectiveness of orthopaedic procedures through multiple mechanisms. These include avoidance of the costs of preoperative medical clearance and anesthesiologist fees,^2,8,9^ reductions in surgical supply costs, and avoidance of the high overhead costs associated with personnel and equipment required for standard operating rooms (ORs), as many procedures can be performed in the procedure room setting.^10^ Historically, the primary resistance to the adoption of WALANT in hand surgery was the dogmatic view that injection of epinephrine into the finger commonly led to tissue necrosis. However, a series of studies have disproved this theory,^6,11,12,13,14^ including a seminal study of 3100 consecutive cases of elective finger and hand surgeries using low-dose epinephrine by nine surgeons in six cities, which demonstrated no incidences of finger infarction and no need for epinephrine reversal using phentolamine.^11^ In addition to hand surgery, WALANT techniques have been successfully used in the treatment of foot and ankle,^1,3,15^ midshaft ulna,^16^ and distal radius fractures.^17,18^

Since first cases of COVID-19—the illness caused by SARS-CoV-2—were reported in December 2019, a global pandemic that is overwhelming healthcare systems around the world has ensued.^19-21^ In an effort to conserve critical healthcare physical and human resources and protect patients, the Centers for Medicare and Medicaid Services, in collaboration with the American College of Surgeons, published recommendations to postpone nonessential surgeries and procedures during the outbreak.^22,23^ Using a three-tier system (with two subclasses within each tier), the recommendations suggest that cases be classified as tier 1—low acuity surgeries for non–life-threatening illnesses that should be postponed, tier 2—intermediate acuity surgeries for non–life-threatening illnesses with potential for future morbidity and mortality that should be postponed if possible, or tier 3—high acuity surgeries that should not be postponed.^22,23^ In alignment with these guidelines, the American Academy of Orthopaedic Surgeons suggests that surgical triage decisions be made by multidisciplinary teams at an institutional level to accommodate variability in resource availability at the local level.^24^

To further mitigate the effect of tier 2 procedures on scarce resources such as personal protective equipment (PPE), ventilators, and anesthesiology providers, our orthopaedic service began routine use of WALANT rather than standard general or local anesthesia, whenever possible.

We present a case series of our early experience with a service-wide implementation of WALANT in 16 orthopaedic procedures during the COVID-19 pandemic.

## Methods

This study was deemed institutional review board exempt as a retrospective review of a quality improvement initiative. All patients undergoing orthopaedic surgery with WALANT during April 2020 at a single regional medical center were included in this case series. Surgeries were performed at the medical center's main OR or at the institution's ambulatory surgery center. Case details were abstracted from the electronic medical record, and descriptive statistics were performed using SPSS version 26 (IBM). The primary end point was WALANT failure, defined as intraoperative conversion to general anesthesia. Secondary end points included OR time, recovery room time, and pain scores and narcotic consumption in the recovery room.

No WALANT surgeries had been performed at our institution before this series of cases. Before initiation of the first case, all surgeons participated in a web-based training conducted by an experienced WALANT orthopaedic surgeon. Patients were prospectively identified as potential candidates for inclusion based on the presence of a musculoskeletal extremity injury or condition requiring surgical intervention that the surgical team believed could effectively and safely be managed by the direct infiltration of local anesthesia with epinephrine (eg, tumescent anesthesia). Anxiety and mental status were secondary considerations as to whether the patient would tolerate the technique. When considering the use of WALANT, the operative surgeon weighed the potential risks and benefits of the technique. The most notable benefits were considered to include elimination of sedation and its associated adverse effects, decreased need for medical clearance, elimination of the need for preoperative fasting, elimination of tourniquet pain, and elimination of anesthesia induction time. The most notable risks considered were decreased operative field visualization from a lack of a tourniquet, failure to control intraoperative pain, and tissue necrosis from epinephrine injection. Because of the risk of tissue necrosis, patients with hypersensitivity to lidocaine or peripheral circulation conditions were not considered candidates for WALANT. These risks and benefits must be considered on patient-specific basis and in the context of the clinical resources available at the time of surgery. All patients were provided an explanation of the risks and benefits of WALANT and thoroughly educated on what to expect during the surgical experience. For 10 cases, anesthesiologist support and full anesthesia equipment were present to facilitate a rapid induction of general anesthesia if required. Low-dose intravenous sedation was used in some cases with anesthesiologist support at provider discretion. In addition to the primary surgeon, one assisting surgeon scrubbed each case to facilitate procedure efficiency and engage with the awake patient as necessary.

A standard WALANT solution consisting of 50 mL of 1% lidocaine, 50 mL of injectable normal saline, 10 mL of 8.4% bicarbonate, and 1 mL of 1:1,000 epinephrine was used for all cases. Weight-based dosing of a maximum of 7 mg/kg of lidocaine with epinephrine was adhered to.^2,25^ Injection into the appropriate anatomical location was performed using a 27-gauge needle slowly over 10 to 20 minutes and occurred 45 to 60 minutes before incision. Any remaining solution not injected before incision was injected intraoperatively if additional analgesia was required.

## Results

A total of 16 WALANT cases were performed by 7 surgeons with fellowship training in sports medicine; hand, upper extremity, and microvascular surgery; and total joint arthroplasty. Of the patients undergoing surgery with WALANT, 56% were female, 63% were White, and the average age was 41 years with an average body mass index of 29.2 kg/m^2^. Nineteen percent of patients had an American Society of Anesthesiologists (ASA) classification of 1 at the time of surgery, whereas 31% were ASA 2 and 13% were ASA 3 (Table 1).

**Table 1:** Patient Demographics

Patient Demographics	Average (Min-Max) or Number (% of Patients)
Total patients	16 (100)
Female	9 (56)
White	10 (63)
Age (yrs)	41 (19-71)
Body mass index (kg/m^2^)	29.2 (15.9-40.3)
ASA class	
None documented	6 (37)
1—Healthy	3 (19)
2—Mild systemic disease	5 (31)
3—Severe systemic disease	2 (13)

ASA = American Society of Anesthesiologists

Indications for surgery and the procedures performed were variable and addressed pathologies across the hand, upper extremity, thigh, knee, ankle, and foot (Table 2). Anesthesiologist backup was present for 63% of cases. Surgical time averaged 83 minutes with a range of 30 to 166 minutes. No instances of WALANT failure requiring conversion to general anesthesia occurred. In recovery, one patient (6%) required narcotics for pain control, and the average postoperative pain numeric rating scale was 0.6. The maximum pain score experienced was four in the patient requiring postoperative narcotics. The average time in recovery was 42 minutes and ranged from 8 to 118 minutes (Table 3).

**Table 2:** Diagnoses and Surgical Procedures Performed

Sequential Case No.	Diagnosis	Surgical Procedure
1	Metadiaphyseal fracture of the right radial shaft	ORIF (volar plate)
2	2 fragment distal radius fracture	ORIF (volar plate)
3	Unstable trimalleolar equivalent ankle fracture with syndesmotic injury	ORIF (fibular plate) and syndesmotic stabilization
4	Carpal tunnel syndrome and ulnar neuropathy of the upper extremity	Carpal tunnel and cubital tunnel release
5	Closed displaced fracture of the clavicle	Superior plating with augmented suture cerclage of comminuted fragments
6	Complete quadriceps tendon rupture	Quadriceps tendon repair
7	Distal radius metadiaphyseal fracture	Percutaneous pinning
8	Ulnar neuropathy	Cubital tunnel release
9	Intra-articular fracture of the distal radius	ORIF (volar plate)
10	Displaced third and fourth metacarpal shaft fractures	ORIF (lag screws)
11	Severe retracted quadriceps tendon rupture	Repair and reconstruction of the quadriceps tendon rupture with V-Y advancement
12	Closed displaced comminuted fracture of the patella	ORIF (cannulated screw and wire)
13	Small finger FDP and FDS laceration and radial digital nerve laceration	Small finger flexor tendon and digital nerve repair
14	Septic olecranon bursitis	Excision of the bursa
15	Thigh and knee abscess with cellulitis	Irrigation and débridement of a deep abscess, right thigh/knee
16	Open extensor tendon laceration	Central slip boutonniere repair

FDP = flexor digitorum profundus, FDS = flexor digitorum superficialis, ORIF = open reduction and internal fixation

**Table 3:** Case Characteristics and Perioperative Outcomes

Outcome Measure	Average (Min-Max) or Number (% of Patients)
Anesthesiologist backup provided	10 (63)
WALANT failure	0 (0)
Minutes in the operating room	83 (30-166)
Narcotics used in the recovery room	1 (6)
Pain NRS in the recovery room	0.6 (0.0-4.0)
Recovery time (min)	42 (8-118)

NRS = numeric rating scale, WALANT = wide-awake local anesthesia no tourniquet

**Figure 1:**
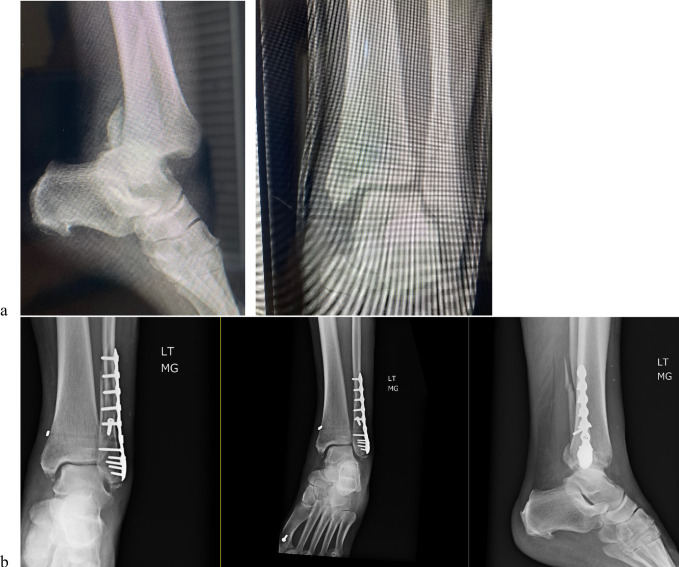
Preoperative and postoperative imaging of unstable trimalleolar equivalent ankle fracture with syndesmotic injury. Preoperative imaging presented in (**A**) demonstrates trimalleolar ankle fracture with syndesmotic injury. (**B**) Postoperative imaging after open reduction and internal fixation with 3.5 × 22-mm lag screw placed perpendicular to the fracture angle, with a 6-hole distal fibular locking plate placed and fit to the lateral aspect of the distal fibula. The patient had a positive intraoperative cotton test for widening. The syndesmosis was reduced, and a tightrope was drilled across the fibula with the supplied guide parallel to the ankle joint. Medially, suture repair of the deltoid ligament and medial malleolar fracture through the tibial periosteum was performed. LT = left, MG = radiology tech.

**Figure 2:**
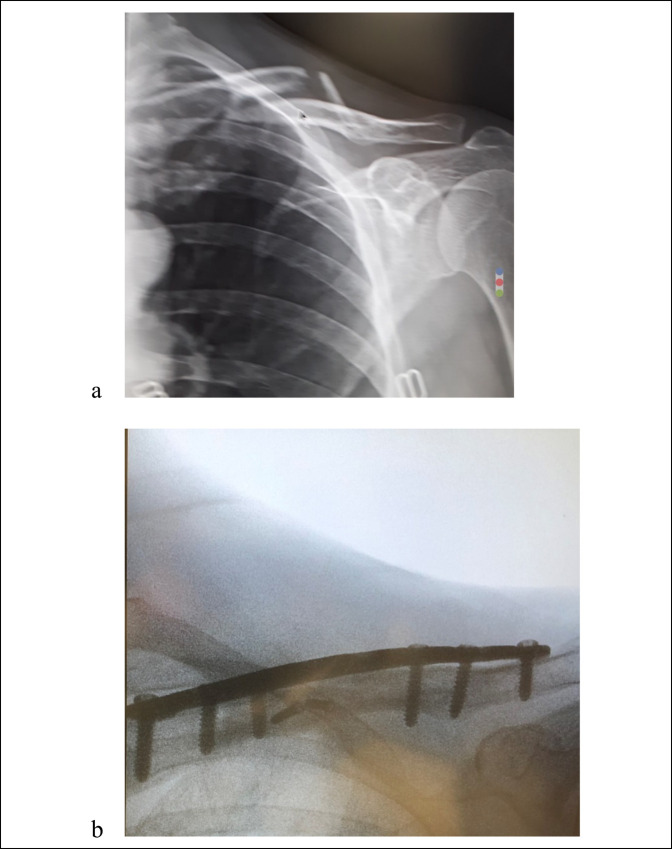
Preoperative and postoperative imaging of closed displaced fracture of the clavicle. Preoperative imaging presented in (**A**) demonstrates closed displaced fracture of the clavicle. (**B**) Postoperative imaging after open reduction and internal fixation with clavicle plate plus interfragmentary compression of the main fracture fragments. A butterfly piece was cerclaged using #2 FiberWire.

This case series includes four notable surgeries for which WALANT techniques have not been previously been described. These include an open reduction and internal fixation (ORIF) for a closed displaced fracture of the clavicle, ORIF for a closed displaced comminuted fracture of the patella, and two quadriceps tendon repairs for complete quadriceps tendon ruptures. On confirmation of successful analgesia, all cases were performed using standard techniques. None of these four cases required postoperative narcotics in recovery, and only one patient undergoing quadriceps repair reported a postoperative pain numeric rating scale (NRS) greater than zero (NRS = 2). Preoperative and postoperative imaging of notable fracture cases is presented in Figures 1 and 2, and video depicting the tumescent anesthesia injection technique, intraoperative patient evaluation, and radiographic results is presented in the supplemental content (http://links.lww.com/JG9/A107).

## Discussion

Our results demonstrate that WALANT techniques can be safely and effectively used to treat a broad array of orthopaedic pathologies and can be applied in both soft tissue and bony procedures. To our knowledge, this is the first published description of WALANT surgery used to perform ORIF of a clavicle fracture, ORIF of a patella fracture, or for repair of a ruptured quadriceps tendon. Most importantly, this case series presents a paradigm for allowing access to orthopaedic surgical care during times of disaster that impose notable limits on medical resources.

In response to the COVID-19 pandemic, the orthopaedic surgery community has demonstrated the ability to innovate new approaches to caring for patients that provide protection to both patients and providers and allow for effective allocation of scarce resources. Across specialties, the speed with which providers have transitioned to telemedicine to effectively deliver care has been remarkable, as evidenced by the increase from 18% of physicians conducting telemedicine visits in 2018 to 48% in April 2020.^26^ Rapid development and dissemination of orthopaedic protocols for physical examination using innovative techniques such as online goniometers and the use of common household objects with standard weights to assess strength has occurred to maximize the effectiveness of these virtual visits.^27^ To address PPE shortages, orthopaedic surgeons from Duke University retrofitted arthroplasty helmets with a novel 3D-printed manifold, and other modifications provide a possible solution to the shortage of powered air-purifying respirators.^28^ Aside from these innovations, the successful redeployment of orthopaedic surgeons to treat COVID-positive patients in the medical wards, intensive care units, and emergency departments is a demonstration of the ability to rapidly evolve skill sets and operationalize new models of care delivery.^29^

Although WALANT techniques are well described in hand surgery and beginning to proliferate for use in other orthopaedic procedures, we suggest the rapid roll out of service-wide WALANT program in response to OR resource constraints marks a notable innovation by orthopaedic surgeons. Given the shortage of ventilators and anesthesia providers available during the COVID-19 pandemic, this technique has enabled independent performance of cases by orthopaedic surgeons that would not be otherwise possible because of resource constraints. Although this case series incorporated anesthesiologist backup in 10 of 16 cases during the pilot phase, continued use of WALANT at our institution will not include anesthesia support. Other potential benefits that will be formally evaluated upon full program roll out include total OR time savings from elimination of anesthesia induction, postoperative pain scores in comparison with the use of a peripheral block with a tourniquet, economic impact of reduced anesthesia personnel, drugs and equipment, and the potential savings realized from reducing the need for postanesthesia care unit physical and human resources. Use of this technique, paired with adoption of reusable gowns and drapes, presents a sustainable means for providing orthopaedic surgical care when faced with anesthesiologist, ventilator, and PPE shortages. We suggest that familiarization with WALANT be included in future disaster preparedness training for orthopaedic departments to facilitate rapid implementation of these techniques should future need arise.

Beyond times of disaster or in resource-constrained environments, the benefits of WALANT make it attractive for increased adoption into routine orthopaedic practice. In Canada, the technique is used in over 90% of carpal tunnel releases. Its use has resulted in notable cost savings, as these procedures can be performed four times cheaper and twice as efficiently in the ambulatory room rather than OR setting.^2,30^ In their description of the Canadian model for hand surgery, Wheelock et al.^31^ highlight the national adoption of wide-awake techniques that, in addition to providing direct cost savings, have freed precious resources such as ORs and hospital beds for high acuity procedures. Given the unsustainable trend in health care spending in the United States, and the fact that musculoskeletal conditions accounted for $190 billion of medical service expenditures in 2013, increased use of WALANT may enhance the value of orthopaedic surgical care.^32^ Alternatively, low- and middle-income regions of the world routinely face resource constraints similar to those imposed on developed countries during the COVID-19 pandemic.^33^ With nearly five billion people around the world lacking access to safe and affordable surgical care,^34^ expanded uptake of WALANT could enhance access in these low-resource environments.

As a case series, this study has several limitations. Our single-institution review of a small sample size exposes this study to selection bias and may limit the generalizability of our findings. Second, our evaluation of postoperative pain scores in the PACU only limits our ability to evaluate pain levels after the local anesthetic has worn off. Further study is required to evaluate whether WALANT results in higher levels of early postoperative pain during the 4 to 6 hours after surgery once the effect of local anesthetic has dissipated, in comparison with standard anesthetic techniques. Third, no comparison to a control cohort of patients undergoing surgery with general or regional anesthesia is presented, and long-term radiographic, clinical, or patient-reported outcomes were not assessed. Before wide spread adoption of WALANT for orthopaedic procedures outside of hand surgery, higher quality prospective studies must be conducted.

## Conclusion

In response to OR resource constraints imposed by the COVID-19 pandemic, our orthopaedic service rapidly adopted and expanded its use of WALANT techniques. The approach was safely and effectively used in 16 cases across multiple orthopaedic subspecialties, including three procedures not previously described in the literature. WALANT techniques hold promise for use in future disaster scenarios and should be evaluated for potential incorporation into routine orthopaedic surgical care.
